# Insights into *Alexandrium minutum* Nutrient Acquisition, Metabolism and Saxitoxin Biosynthesis through Comprehensive Transcriptome Survey

**DOI:** 10.3390/biology10090826

**Published:** 2021-08-25

**Authors:** Muhamad Afiq Akbar, Nurul Yuziana Mohd Yusof, Fathul Karim Sahrani, Gires Usup, Asmat Ahmad, Syarul Nataqain Baharum, Nor Azlan Nor Muhammad, Hamidun Bunawan

**Affiliations:** 1 Department of Biological Sciences and Biotechnology, Faculty of Science and Technology, Universiti Kebangsaan Malaysia, Bangi 43600, Malaysia; muhdafiq.akbar@gmail.com (M.A.A.); asmat@ukm.edu.my (A.A.); 2 Department of Earth Science and Environment, Faculty of Science and Technology, Universiti Kebangsaan Malaysia, Bangi 43600, Malaysia; yuziana@ukm.edu.my (N.Y.M.Y.); fathul@ukm.edu.my (F.K.S.); gires@ukm.edu.my (G.U.); 3 Institute of System Biology, Universiti Kebangsaan Malaysia, Bangi 43600, Malaysia; nataqain@ukm.edu.my (S.N.B.); norazlannm@ukm.edu.my (N.A.N.M.)

**Keywords:** *Alexandrium minutum*, dinoflagellates, harmful algae blooms, saxitoxin, transcriptomics

## Abstract

**Simple Summary:**

*Alexandrium minutum* is one of the causing organisms for the occurrence of harmful algae bloom (HABs) in marine ecosystems. This species produces saxitoxin, one of the deadliest neurotoxins which can cause human mortality. However, molecular information such as genes and proteins catalog on this species is still lacking. Therefore, this study has successfully characterized several new molecular mechanisms regarding *A. minutum* environmental adaptation and saxitoxin biosynthesis. Ultimately, this study provides a valuable resource for facilitating future dinoflagellates’ molecular response to environmental changes.

**Abstract:**

The toxin-producing dinoflagellate *Alexandrium minutum* is responsible for the outbreaks of harmful algae bloom (HABs). It is a widely distributed species and is responsible for producing paralytic shellfish poisoning toxins. However, the information associated with the environmental adaptation pathway and toxin biosynthesis in this species is still lacking. Therefore, this study focuses on the functional characterization of *A. minutum* unigenes obtained from transcriptome sequencing using the Illumina Hiseq 4000 sequencing platform. A total of 58,802 (47.05%) unigenes were successfully annotated using public databases such as NCBI-Nr, UniprotKB, EggNOG, KEGG, InterPRO and Gene Ontology (GO). This study has successfully identified key features that enable *A. minutum* to adapt to the marine environment, including several carbon metabolic pathways, assimilation of various sources of nitrogen and phosphorus. *A. minutum* was found to encode homologues for several proteins involved in saxitoxin biosynthesis, including the first three proteins in the pathway of saxitoxin biosynthesis, namely sxtA, sxtG and sxtB. The comprehensive transcriptome analysis presented in this study represents a valuable resource for understanding the dinoflagellates molecular metabolic model regarding nutrient acquisition and biosynthesis of saxitoxin.

## 1. Introduction

Dinoflagellates have a large genome size of up to 245 Gbp and over 100 chromosomes [1]. This represents one to 80 times the size of the human haploid genome. The size of the dinoflagellate genome also differs from many other eukaryotic algae, whose genome size is usually between tens to hundreds of Mbp [2]. This unique feature and unusual genome size is a significant barrier to large-scale genomic studies of these organisms [3,4]. To date, the only dinoflagellate genome that has been successfully sequenced is limited *Symbiodinium* spp., *Amphidinium gibbosum*, *Amoebophrya ceratii* and *Polarella glacialis* due to the smallest size genome among dinoflagellates [5,6,7,8,9]. The first draft genome of *Symbiodinium minutum* has been successfully sequenced by Shoguchi et al. [10] using the Roche 454 GS-FLX platform and Illumina Genome Analyzer IIx (GAIIx) next-generation sequencers, which assembled a total of 616 Mpb of genome out of a total of 1.5 Gpb of total genome size predicted for the species. However, dinoflagellates of the genus Symbiodinium are dinoflagellates in symbiosis with corals and do not produce saxitoxin or trigger the phenomenon of harmful algae blooms (HABs) [11]. Thus, the mechanisms for dinoflagellate toxin biosynthesis, life cycle regulation and response to environmental changes at the molecular level remain poorly understood due to the lack of genome and gene information involved in the process [1,3].

A transcriptomic approach using a next-generation sequencing platform, such as Illumina Hiseq, is an alternative for studying organisms with complex genomes such as dinoflagellates [12]. The transcriptomic approach is an ideal and holistic approach to analyze the mRNA sequences expressed by cells at specific times and conditions. There are existing transcriptomic data of several dinoflagellate species contributing to the phenomenon of HABs including both toxic (*Alexandrium minutum*, *Alexandrium fundyense*, *Alexandrium catenella*, *Prorocentrum minimum* and *Karenia brevis*) and non-toxic (*Alexandrium tamutum*, *Prorocentrum donghaiense*, *Scrippsiella trochoidea*, *Cochlodinium polykrikoides*, *Amphidinium carterae*, *Prorocentrum micans*) strains have been reported using transcriptomic approach via next-generation sequencing platform [3,13,14,15,16,17,18,19,20]. These studies have allowed the discovery of dinoflagellates’ response toward stressors and determining putative genes responsible for toxin biosynthesis.

In this study, the next-generation sequencing platform Illumina Hiseq 4000 was used to generate transcriptome data of *A. minutum*. To obtain transcriptome sequence data relevant to the study questions involving the effects of nutrient changes, sequence data were generated from *A. minutum* cultured in normal environment, nitrogen-deficient environment and phosphorus-deficient environment. Several major components in the cellular metabolism of *A. minutum,* as well as homologues for the saxitoxin biosynthesis gene will be characterized. This study also additional insights into the gene catalog encoded by *A. minutum* along with detailed annotations. The information generated from this study is an important component to understand the mechanism of response to environmental changes as well as the biosynthesis of saxitoxin for *A. minutum*.

## 2. Materials and Methods

### 2.1. Cell Culturing and Harvesting

The dinoflagellate culture of *A. minutum* AmKB01 used throughout this study was obtained from the culture collection of the Marine Microbiology and Biotechnology Laboratory, National University of Malaysia. This strain was isolated from the mangrove waters in Tumpat, Kelantan, in 2001 when an outbreak of shellfish poisoning in the area caused one death, and several people were hospitalized. The production of saxitoxin by this strain has been determined by previous studies [21]. The growth medium for *A. minutum* used in this study was ES-DK media added to seawater with a salinity of 15 psu [22]. The cultures were maintained at 26 °C at 12:12 h dark: light regime with an irradiance of 100 µmol photons m^−2^ s^−1^ light intensity. To include genes expressed under different physiological conditions, A. *minutum* cells were subjected to nitrogen and phosphorus limitation for 72 h before harvesting. In order to induce nitrogen/phosphorus limitation, *A. minutum* cells were cultured in standard ES-DK media first until they reached the exponential phase before being transferred to new ES-DK media without nitrogen/phosphorus component using a 15 µm mesh sieve. The absence of nitrogen/phosphorus in the culture media was confirmed using a HACH DR2800 spectrophotometer (HACH, Ames, IA, USA) [23].

### 2.2. RNA Extraction

Harvested *A. minutum* cells were frozen immediately in liquid nitrogen and stored at −80 °C until RNA extraction. RNA extraction was performed using Trizol reagent (Sigma-Aldrich, St. Louis, MO, USA) as per the manufacturer’s protocol. The concentration and purity of the extracted RNA were determined using a Nanodrop-1000 Spectrophotometer machine (Thermo Fischer Scientific, Waltham, MA, USA) through readings at 260 nm and 280 nm absorption. An absorption reading ratio of 260:280 nm around 1.8 to 2.0 was considered good. Finally, RNA integrity was also measured using a 2100 Bioanalyzer machine (Agilent Technologies, Santa Clara, CA, USA). Intact RNA would show a RIN score above 7. Only RNA samples that show the quality meets the standards would be used for RNA sequencing. Two biological replicates from each treatment group were used for sequencing.

### 2.3. cDNA Library Construction and Illumina Sequencing

According to the manufacturer’s protocol, the cDNA library preparation was conducted using SureSelect Strand-Specific RNA Library Prep (Agilent Technologies, Santa Clara, CA, USA). The sequencing of cDNA libraries was performed using the Illumina Hiseq4000 150PE (Theragen Etex, Suwon, Gyeonggi, Korea) platform.

### 2.4. Transcriptome Assembly

Raw data reads for all samples generated from Illumina sequencing were pre-processed using the Trimmomatic (v0.36) [24]. Next, high-quality raw data reads were assembled de novo via Trinity (v2.8) [25]. The Trinity program was run with modifications on the following parameters namely min_kmer_cov = 2 and SS_lib_type = RF. The expression level of each transcript assembled was calculated using the RSEM [26]. Transcripts that show an expression level of less than 1 FPKM are considered artifacts and are then discarded using the “filter_low_expr_transcripts.pl” script from the Trinity package. After that, the script from the Trinity package “get_longest_isoform_seq_per_trinity_gene.pl” was used to extract the longest representative for each transcript that will be called unigenes. These unigenes were then used in further analysis throughout this study.

### 2.5. Quality Evaluation of Transcriptome Assemblies

This study used two methods to evaluate the quality of the *A. minutum* assembled unigenes: assessing the percentage of raw data mapping to the transcriptome assemblies and based on the conserved orthologue sequence content [27]. The HISAT2, a fast and sensitive alignment program for mapping sequence reads to transcriptome data sets, is used to evaluate the percentage of raw data re-mapping [28]. Next, the presence of preserved orthologous sequence content was assessed using the BUSCO based on the Eukaryotic data set (odb9) [29]. In addition, the “contig_ExN50_statistic.pl” script from the Trinity package was used to compute the ExN50 profile of our assembled unigenes.

### 2.6. Prediction of the Protein-Coding Sequence

Protein-encoding sequences were identified from *A. minutum* unigenes using the TransDecoder program [25]. Uniprot and Pfam-A databases were used to maximize the accuracy of the ORF sequence identification.

### 2.7. Transcriptome Annotation

The unigenes sequences were annotated against protein databases, NCBI non-redundant protein (NCBI-Nr) and UniprotKB (SwissProt and TrEMBL) using the blastx program with e-value parameter ≤ 0.00001 [30]. Next, the InterProScan 5 was used to predict the domain for the identified ORF of *A. minutum*. The program identified homologies for protein domains compiled from 15 databases (Phobius, TMHMM, Pfam, ProDom, Gene3d, Panther, SuperFamily, Coils, SMART, PrositeProfiles, PRINTS, SignalP, PIRSF, TIGRFAMs and HAMAP) [31]. The classification of ORF sequences *A. minutum* to ortholog protein groups was carried out using EggNOG 5.0 [32]. Finally, the annotation of *A. minutum* unigenes to the biochemical pathway from the Kyoto Encyclopedia of Genes and Genomes (KEGG) database was performed using the GhostKoala program [33]. Subcellular localization of proteins was predicted bioinformatically through the BUSCA webserver [34].

**Figure 1:**
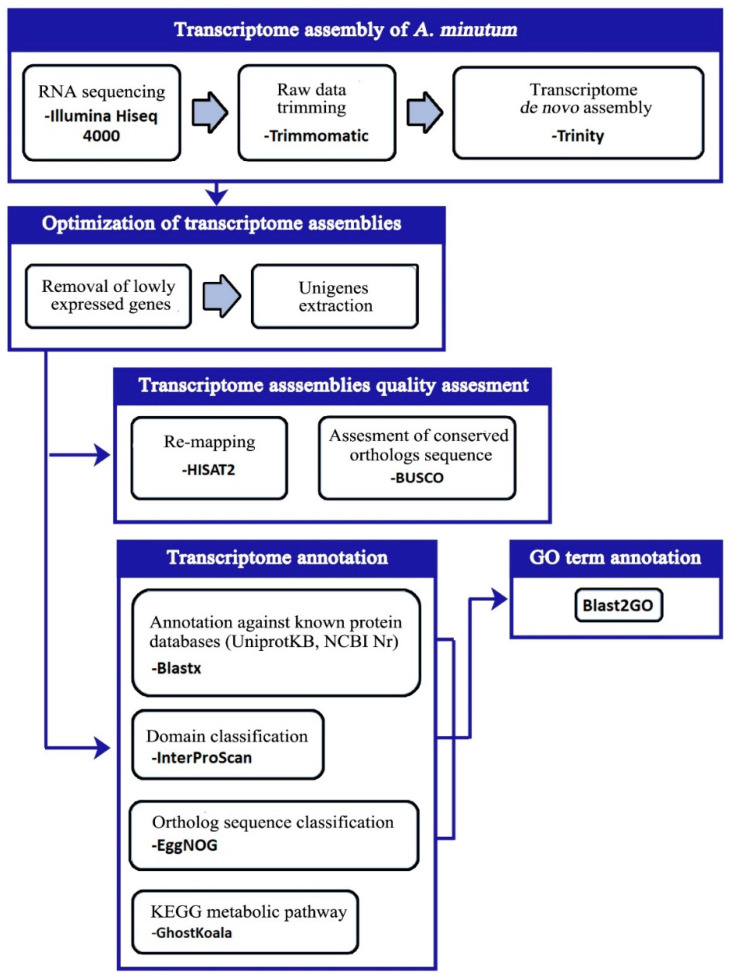
Flow chart of *A. minutum* transcriptome analysis method used in this study.

The Blast2GO 5.2 program was used to define gene ontology (GO) terms for each unigenes in the *A. minutum* transcriptome set [35]. The GO term is filtered using the parameter “permissive annotation” to remove the incorrectly matched GO term. To determine additional GO terms for unigenes in this study, the Blast2GO 5.2 program was also used to combine GO terms from the InterPRO and EggNOG matching results to the existing GO terms. Figure 1 displays the flow chart for the transcriptome analysis method in this study.

### 2.8. Identification of Putative Saxitoxin Genes

With reference to the pathway of saxitoxin biosynthesis by Kellman et al. [36], saxitoxin biosynthesis protein sequences from cyanobacteria were downloaded from the NCBI Nr database as FASTA format. Next, the “makeblastdb” function of the Blast+ program was used to generate a protein database consisting of cyanobacterial saxitoxin biosynthesis proteins [30]. Finally, the blastx program was used to match the *A. minutum* unigene sequence to a newly constructed cyanobacterial saxitoxin biosynthesis protein database with a parameter value of e ≤ 0.00001.

## 3. Results and Discussions

### 3.1. Transcriptome Sequencing and De Novo Assembly

The *A. minutum* transcriptome cDNA library was generated from six biological samples induced by three different environments (control, nitrogen deficiency, and phosphorus deficiency) using Illumina Hiseq 4000 platform sequencing platform. A total of 295.4 million high-quality readings with base readings of 40.63 Gpb were successfully produced (Supplementary Material Table S1). Next, de novo assembly using Trinity and filtering of the transcript with an expression value below 1 FPKM produced a total of 124,977 final unigenes for *A. minutum* to be used in downstream analysis. The unigenes produced comprised 112.07 Mbp, had a value of N50 at 1566 bp and the average unigenes length was at 896.69 bp (Table 1). Moreover, almost 50% of the assembled unigenes have lengths exceeding 500 bp.

To date, there is still no gold standard method for determining the quality of transcriptome assemblies for non-model organisms such as dinoflagellates. This study takes the approach suggested by Tong et al. [27]. To evaluate the quality of the *A. minutum* unigenes, the raw reads were mapped back to the assembled unigenes using HISAT2, and the results showed that 80% of the total reads were mapped correctly. The completeness of *A. minutum* unigenes was assessed based on the ortholog content maintained through the BUSCO based on the Eukaryotic data set (odb9) [29]. As a result, 83.5% of the 303 BUSCO orthologues (71.0% complete; 12.5% fragmented) were identified from *A. minutum* unigenes. In addition, the ExN50 profile of our assembled unigenes peaked at 91% of the total expression indicating an adequate sequencing depth (Supplementary Material Table S2) [37].

### 3.2. Coding Region Prediction

The TransDecoder was used to predict ORF sequences and protein sequence translation from the assembled unigenes. UniProtKB and Pfam databases were used to help identify ORFs. A total of 67,093 unigenes (53.68% of the total unigenes) were predicted to encode ORFs with an average value of 419 amino acids and an N50 value of 536 amino acids. Among the predicted ORFs, a total of 44,376 ORFs contained start codons and end codons, indicating that these ORFs were present as ORFs with a complete sequence. ORF sequences were used to perform annotation against KEGG, EggNOG and InterPro databases.

### 3.3. Unigenes Annotation

**Figure 2:**
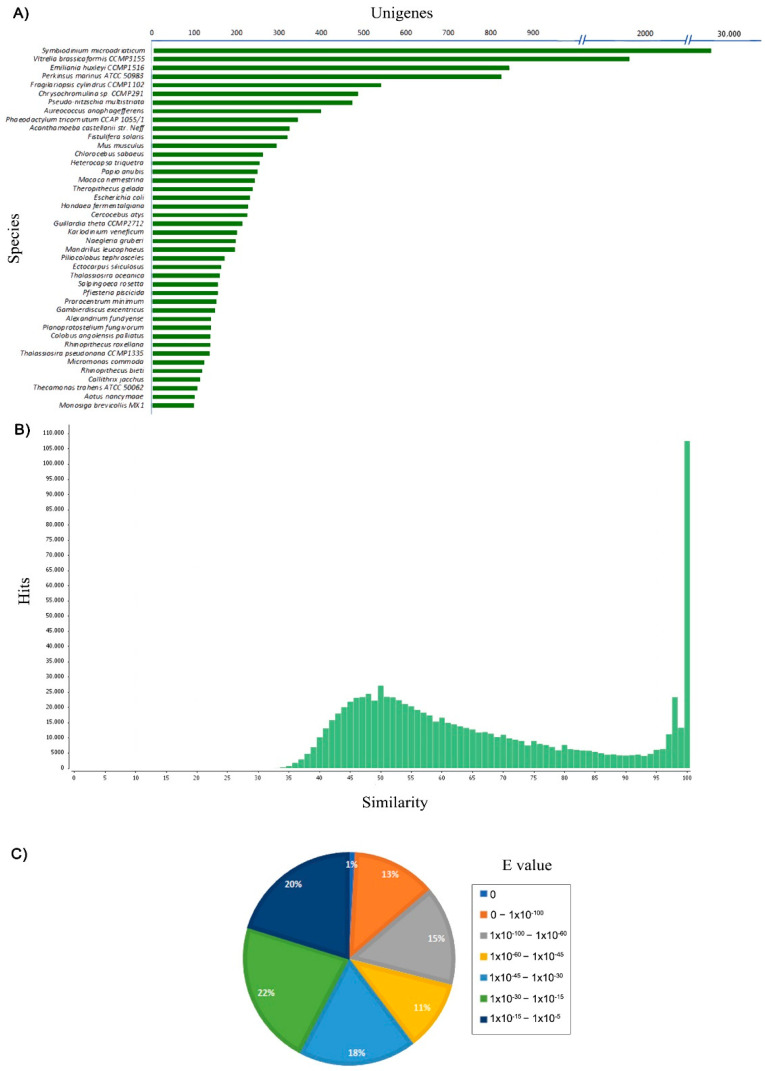
Annotation of *A. minutum* unigenes against the NCBI-NR database. (**A**) The homological species distribution for *A. minutum*. (**B**) The percentage of similarity of sequences identified using blastx program and (**C**) the distribution of E values of the blastx hit.

To determine the function of the assembled unigenes, all unigenes were compared to the sequences available in public databases such as the NCBI Nr, UniProtKB, KEGG, InterPro, EggNOG and GO databases. Of the 124,977 unigenes compared against the NCBI Nr database, only 58,802 (47.05%) unigenes produced significant matches, while the InterPro database annotation results recorded a total of 57,605 (46.09%) significant matches (Table 2). A total of 70% (27,552 unigenes) of the unigenes annotation results against the NCBI Nr database showed homology with proteins from dinoflagellate *Symbiodinium microadriaticum* (Figure 2A). Other matches with the NCBI Nr database were also included proteins from other marine phytoplankton groups, such as *Vitrella brassicaformi* (1927 unigenes), *Emiliania huxleyi* (846 unigenes), *Perkinsus marinus* (827 unigenes), *Fragilariopsis cylindrus* (544 unigenes) and *Chrysochromulina* sp. (489 unigenes). Protein similarity distribution shows that 47.5% of unigenes sequences had similarities higher than 60%, and 52.5% of unigenes sequences showed similarity between 33% and 60% to known protein (Figure 2B). According to the distribution of expected e values from the top matches based on the NCBI Nr database, 40% of the matched unigenes sequences showed high homology (<1 × 10^−45^), while 60% of the matched sequences showed moderate homology (between 1 × 10^−5^ and 1 × 10^−45^) (Figure 2C). Overall, these results indicate that *A. minutum* assembled unigenes maintains a level of conservation with known proteins.

More than 50% of the assembled unigenes did not show any match with any of the protein sequences found in the NCBI Nr and UniprotKB databases. The low annotation success rate is primarily due to the very limited number of dinoflagellate reference genomes. To date, available dinoflagellate genome data consists only of symbiotic or parasitic species except for *A. gibbosum* [6,7,8,10,11,38,39,40]. This group was selected for sequencing because of its small genome size, i.e., 0.12–6.4 Gbp [5,8,9]. In comparison, other dinoflagellate genomes are much larger, ranging between 3–245 Gbp [13]. Thus, the matching of dinoflagellate transcriptome data to protein databases such as NCBI Nr and UniprotKB is seen to be highly dependent on sequence sources from other phytoplankton groups. Despite having a low match to the protein sequences found in the NCBI Nr database, the annotation results of our *A. minutum* transcriptomes showed a comparable percentage of annotations than some other recent studies of other dinoflagellate transcriptomes such as *A. minutum* (42.3%), *Amphidinium carterae* (33%), *Prorocentrum donghaiense* (40%), *Scrippsiella trochoidea* (41%), *Yihiella yeosuensis* (47.7%), *Gambierdiscus polynesiensis* (51.8%) and *Gambierdiscus pacificus* (51.1%) [15,41,42,43,44,45].

**Figure 3:**
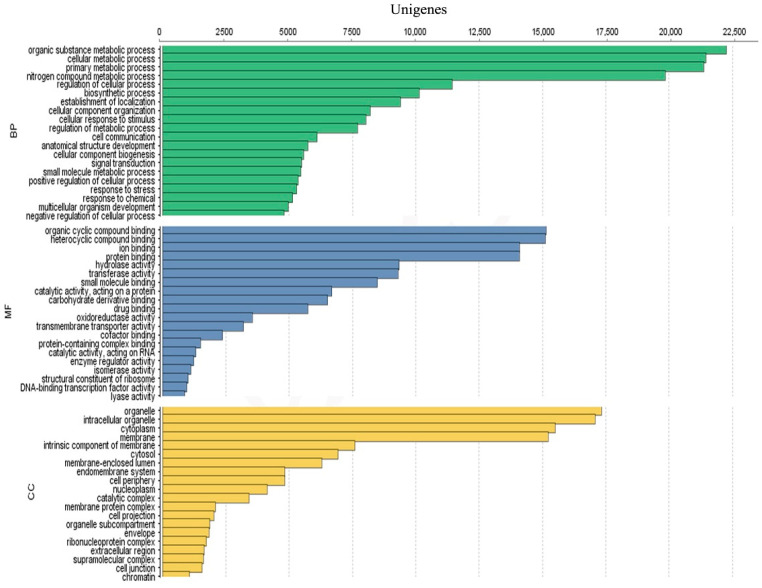
Annotation of GO terms from categories of cellular components (CC), molecular function (MF) and biological processes (BP) for *A. minutum* unigenes. Only the top 20 terms for each category are shown.

Annotated unigenes from NCBI Nr, UniprotKB, EggNOG and Interpro were further assigned to GO terms. A total of 57,128 unigenes were successfully assigned into 17,563 GO terms. For the biological process category, the three main GO terms are “organic substance metabolic process” (GO: 0071704; 22,264 unigenes), “cellular metabolic process” (GO: 0044237; 21,450 unigenes) and “primary metabolic process” (GO: 0044238; 21,365 unigenes). Furthermore, the category of molecular function is dominated by the terms GO “organic cyclic compound binding” (GO: 0097159; 15,150 unigenes), “heterocyclic compound binding” (GO: 1901363; 15,113 unigenes) and “ion binding” (GO: 0043167; 14,913 unigenes). Finally, the category of cellular components is represented by the terms GO “organelle” (GO: 0043226; 17,239 unigenes), “intracellular organelle” (GO: 0043229; 17,075 unigenes) and “cytoplasm” (GO: 0005737; 15,500 unigenes) (Figure 3). Full annotation for *A. minutum* transcriptome are available in Supplementary Material Table S3.

### 3.4. Carbon Metabolism

**Figure 4:**
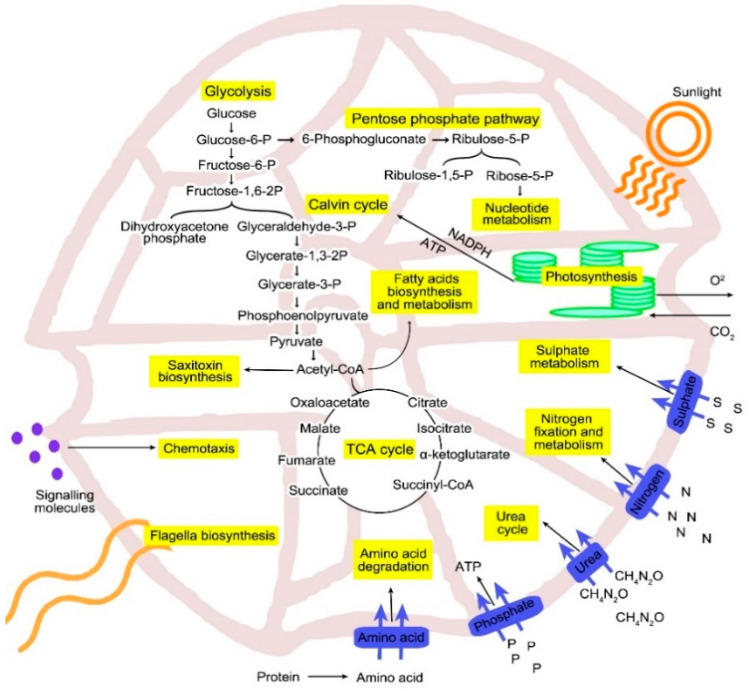
A metabolic pathway model of carbohydrate metabolism and related processes for *A. minutum*.

Carbon degradation is a source of energy metabolism and biosynthetic processes for most organisms [46]. *A. minutum* obtains its carbon source from carbon dioxide and/or organic compounds. Complete genes for core metabolic pathway sites such as Kelvin cycle, tricarboxylic acid cycle (TCA), Embden-Meyerhof-Parnas pathway (EMP or glycolysis) and pentose phosphate pathway (PP) were identified in the *A. minutum* transcriptome (Figure 4). Both EMP and PP pathways are often seen as the backbone of carbon and energy metabolism for eukaryotic organisms, including in phytoplankton such as diatoms [47,48]. These metabolic pathways play an essential role in generating ATP, NAD (P) H and precursors for biosynthetic amino acids, nucleotides, and fatty acids [46].

The diffusion of CO_2_ in the moving marine environment is 5000 times slower than the atmosphere and indirectly limits the uptake of carbon sources by phytoplankton such as dinoflagellates [49]. Thus, in contrast to terrestrial plants that rely on the RubisCO protein as the protein responsible for the carbon fixation process, some phytoplankton such as *Phaeodactylum tricornutum*, *Thalassiosira pseudonana*, *Ostreococcus tauri* and *Micromonas* sp. have developed C4 carbon fixation pathway [50,51,52,53,54]. Based on the transcriptome analysis in this study, *A. minutum* encode complete genes required for the C4 carbon fixation pathway. A study by Gong et al. [55] also found that genes encoding proteins in the C4 carbon fixation pathway site in dinoflagellates showed increased expression levels during the occurrence of HABs, indicating that this pathway may play an important role during HABs.

Apart from identifying all the enzymes required for the expression of the EMP pathway site, which is the most common glycolytic pathway site present in all organisms, the transcriptome analysis of *A. minutum* in this study also successfully identified the homologues gene-encoding phosphoketolase, the enzyme involved in C5 sugar reduction to produce acetyl phosphate in PP pathway [56]. Homologues for this gene in the marine phytoplankton organism have only been identified in the diatom *P. tricornutum* [57].

Moreover, homologues for almost all proteins involved in the Entner-Doudoroff pathway were also identified in the transcriptome of *A. minutum* (except phosphogluconate dehydratase). The Entner-Doudoroff pathway serves as an alternative metabolic pathway to the EMP pathway for the degradation of glucose molecules to pyruvate. This pathway produces lower energy per glucose than EMP pathways but has advantages in terms of lower protein costs and thermodynamic energy [58]. Previously, the Entner-Doudoroff pathway was considered limited to prokaryotic organisms until the discovery of this pathway in diatom *P. tricornutum* by Fabris et al. [57]. Recent studies have also shown a wider distribution of the Entner-Doudoroff pathway, with genomic evidence supporting the presence of these pathways on cyanobacteria, ferns, algae, mosses and plants [46].

Based on transcriptome analysis, *A. minutum* can utilize carbon sources from several metabolic pathways such as EMP, PP, phosphoketolase and Entner-Doudoroff pathways. Several past studies have demonstrated that activation of the different metabolic pathways can produce differences at phenotypic levels, such as activation of virulence factors in some bacteria [59,60]. Since biosynthesis saxitoxin in dinoflagellates also depends on precursors, such as arginine and acetyl-CoA from carbon metabolism, the association between carbon metabolic pathways and saxitoxin biosynthesis is an essential niche of study.

### 3.5. Nitrogen Metabolism

**Figure 5:**
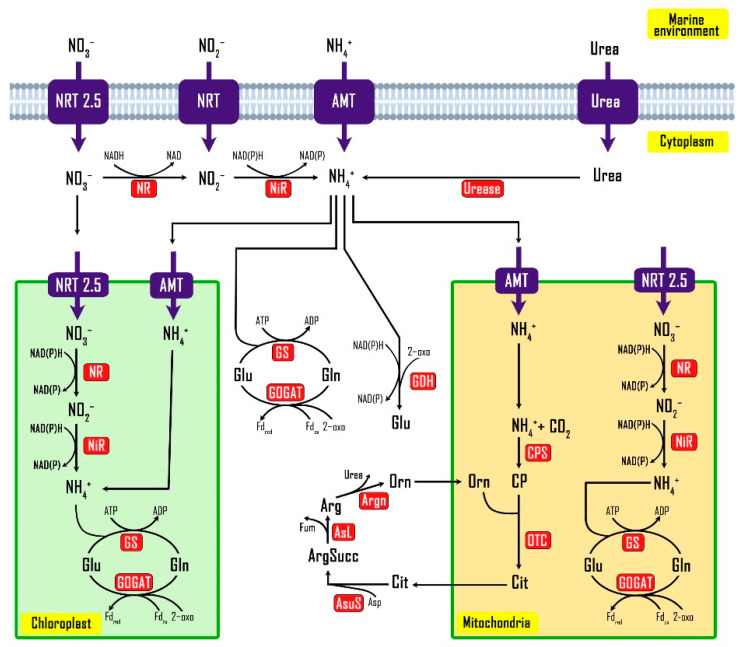
A model for the subcellular localization and metabolic pathway of nitrogen metabolisms by *A. minutum*. The full names of the proteins within the figure legend are available in (Supplementary Material Table S4).

The overall configuration and function of the nitrogen metabolic network for dinoflagellates, including *A. minutum* still receives less attention compared to other model organisms. To assess this more closely, a catalog of genes with potential roles in the assimilation and metabolism of nitrate, urea and ammonia was compiled from the unigenes of *A. minutum*. Subcellular localization of proteins was predicted bioinformatically and used to generate an updated model of nitrogen metabolism in *A. minutum* (Figure 5). Nitrogen metabolism in dinoflagellates is an important branch of study because high nitrogen concentrations often correlate with the phenomenon of HABs and increased rates of saxitoxin production [61,62,63].

Four homologue types of nitrogen transport proteins (nitrate, nitrite, ammonia and urea) were successfully identified in this study. These results are in agreement with several previous studies proving that dinoflagellates of the genus Alexandrium are indeed capable of taking up nitrogen from this source into cells [64,65]. Several recent studies have shown that genes encoding nitrate transporting proteins in *Prorocentrum donghaiense* and *Prorocentrum minimum* were regulated at the transcriptional level and regulated with the changes of nitrogen in the environment [66,67]. Nevertheless, a study by Bellefeuille and Morse [68] found that nitrate transporting proteins in *Lingulodinium polyedrum* were continuously expressed with the presence of nitrogen. Expression of urea transport genes also shows regulation at the transcriptional level and correlates to the presence of urea in the environment [66]. However, there are no detailed studies at the molecular level regarding nitrite uptake by dinoflagellates from the marine environment, although this study successfully demonstrated the presence of gene-encoding nitrite transport proteins by *A. minutum*. This is due to the presence of nitrite in the marine environment that is always at a lower concentration compared to other nitrogen sources [69].

Analysis of the subcellular localization of genes encoding proteins related to nitrogen metabolism showed that the process of nitrate reduction to nitrite and subsequently ammonia occurs in the cytoplasm, mitochondria and even chloroplasts (Figure 5). The GS-GOGAT pathway was predicted to be active in all three of these cell compartments and is responsible for assimilating nitrogen sources that have been reduced to ammonia using the proteins glutamine synthetase (GS) and glutamate synthase (GOGAT). This pathway combines glutamine (Gln) to 2-oxoglutarate using 2 electrons of either Fd-reduction or NADP (H) and produces two molecules of glutamate (Gln) and can regenerate Gln when amino acids and 2-oxoglutarate are needed [70]. Both Glu and Gln are essential components for amino acid metabolism and cellular nitrogen regulation because these molecules act as both nitrogen recipients and donors [71]. This study demonstrated that *A. minutum* encodes a homologue gene for glutamate dehydrogenase (GDH) protein, which may be advantageous for *A. minutum* because ammonia assimilation through the GDH pathway does not require an ATP such as the GS-GOGAT pathway [71].

Our analysis also showed that *A. minutum* encodes all the genes required in the urea cycle pathway site (CPS, carbamoyl phosphate synthase; OTC, ornithine carbamoyltransferase; AsuS, argininosuccinate synthase; AsL, argininosuccinate lyase; Arg, argininosuccinate lyase). The urea cycle pathway site was previously said to be exclusive to metazoan and was not found in green plants and algae until Armbrust et al. [51] found a complete gene for this pathway from the diatom *T. pseudonana*. However, unlike metazoan organisms that use these pathways to convert highly toxic ammonia into urea for excretory processes, diatoms are seen using these pathways as a method to redistribute carbon and nitrogen to TCA and GS-GOGAT pathways [72]. Since *A. minutum* also encodes complete proteins for the TCA and GS-GOGAT pathways, the function of the urea cycle pathways in these dinoflagellates may resemble those of diatoms. In addition to *A. minutum*, homologues for genes involved in the urea cycle pathway site have also been reported in the dinoflagellates of *A. tamarense*, *Gambierdiscus caribaeus* and *P. donghaiense* [70,73,74]

### 3.6. Phosphorus Metabolism

The uptake of inorganic phosphorus sources from the marine environment by phytoplankton organisms such as dinoflagellates is controlled by phosphorus transport proteins on the cell surface [75]. Several homologue genes for phosphorus transport proteins were identified in this study, such as inorganic phosphate transporter from protozoa *Perkinsus marinus* and *Symbiodinium microadriaticum*, low-affinity phosphate transporter from *S. microadriaticum* and PitA phosphate transporter from *Escherichia* coli. However, all homologues of the phosphorus transport proteins identified were low-affinity phosphorus transport proteins. To date, the distribution of high-affinity phosphorus transporting proteins has been observed to be limited in eukaryotic phytoplankton organisms [75]. Lin et al. [75] did not rule out the possibility that eukaryotic phytoplankton organisms, including dinoflagellates, have novel high-affinity phosphorus transport proteins. Given that approximately 50% of the *A. minutum* unigenes has no match with the protein sequence in the public databases, the likelihood that *A. minutum* encoding a novel high-affinity phosphorus transport protein cannot be ruled out.

After the entry of inorganic phosphorus into dinoflagellate cells, this phosphorus will react with adenosine diphosphate (ADP) to produce ATP with the help of ATP synthase protein [76]. Several homologues for this protein were also identified in *A. minutum,* and through further analysis, the protein was placed in three cell compartments, namely chloroplasts, mitochondria and transmembrane. These results were also in agreement with Lin et al. [75], where ATP is the major product of photosynthesis in chloroplasts, a respiratory product in mitochondria and synthesized by the protein rhodopsin in transmembrane. The resulting ATP will then be used in the metabolic pathways in need.

Phytoplankton, including dinoflagellates, can store excess intracellular phosphorus to support cell metabolism and growth as polyphosphates [75,77]. Five homologues for gene encoding the vacuolar transporter chaperone (VTC) protein were identified on the *A. minutum* transcriptome. VTC is a protein responsible for cutting the phosphorus groups from ATP and transferring them into polyphosphate chains [78]. In addition, *A. minutum* also encodes a PPX-like protein that serves to hydrolyze polyphosphate terminal residues to re-release inorganic phosphorus for the use of metabolic pathways in need [75,78].

### 3.7. Saxitoxin Biosynthesis

Through a blastx search with an E value < 0.00001 against a database of saxitoxin biosynthesis proteins obtained from cyanobacteria protein sequence, a total of 216 homologues for this protein were identified in the transcriptome sequence *A. minutum* (Supplementary Material Table S5). These putative proteins include core proteins that are directly involved in the biosynthesis of saxitoxin (sxtA, sxtB, sxtD, sxtG, sxtH/T, sxtI, sxtJ, sxtS, sxtU, sxtV and sxtW), four proteins proposed to be involved in the modification of saxitoxin into related derivatives (sxtL, sxtN, sxtO and sxtX) and proteins for saxitoxin transport (sxtF/M and sxtP). Of these proteins, sxtJ was first identified in dinoflagellates [13]. The results of the sxt protein search on *A. minutum* are shown in Table 3. Our study indicates that *A. minutum* does not encode two of the saxitoxin biosynthesis core proteins (sxtC and sxtK). In fact, to date, no homologue for these proteins has been successfully identified in dinoflagellates [13]. In cyanobacteria, both of these genes encode hypothetical proteins and are predicted to play a regulatory role in the process of saxitoxin biosynthesis [36]. Additionally, homologue for sxtE, sxtQ, sxtR, sxtY and sxtZ proteins were also not identified in this study. Though homologues for sxtR have been identified in *A. minutum* and *A. fundyense* by Stüken et al. [79], homologues for sxtZ have been identified in *A. minutum* by Meng et al. [15]. The variability of sxt genes even among *A. minutum* strains itself might be due to various toxin profiles exhibit among *A. minutum* strains across their biogeographical distributions [16].

Next, the absence of homologues for some sxt proteins in dinoflagellates, including *A. minutum,* might be due to the biosynthesis process of saxitoxin evolving separately in dinoflagellates and cyanobacteria [80]. Thus, saxitoxin biosynthesis proteins from cyanobacteria that do not have any homologues in *A. minutum* or other dinoflagellates are likely to have been replaced by other protein-encoding proteins with similar biochemical activity. A study conducted by Hackett et al. [81] also stated that only proteins involved in the first three steps in the biosynthesis of saxitoxins (sxtA, sxtG and sxtB) from dinoflagellates have phylogenetic relationships with proteins from cyanobacteria, further reinforcing the claim that saxitoxin biosynthesis in dinoflagellates evolved separately and partially with other proteins. Moreover, the results of the study also indicate that most of the putative saxitoxin biosynthetic proteins identified in this study are present with more than one unigenes or isoform. This is likely due to the homologue for sxt genes in *A. minutum* is also involved with other biological processes [82]. Therefore, more in-depth studies are needed to study the actual function of the putative proteins of saxitoxin biosynthesis in dinoflagellates, including *A. minutum*.

## 4. Conclusions

In this study, *A. minutum* transcriptome profile was successfully generated using Illumina Hiseq 4000 platform. The quality and completeness of our assembled unigenes also were carefully evaluated. Extensive annotation against available databases identifies some important unigenes involved in the process of metabolism of carbon, nitrogen and phosphorus for dinoflagellates. In addition, the existence of phosphoketolase and the Entner-Doudoroff pathway in dinoflagellates were discussed for the first time here. Further work on these pathways activities will enable researchers to understand the complex metabolism of carbohydrates for dinoflagellates. Our results also provide insight into saxitoxin biosynthesis of *A. minutum*. The variability of saxitoxin biosynthesis genes among *A. minutum* strains might give rise to different toxin profiles. Although our study managed to identify almost all the saxitoxin biosynthesis “core” genes, a more comprehensive study is needed as most of these genes exist with more than one homologue. To the best of our knowledge, the transcriptome profile of *A. minutum* presented here may be the most comprehensive described with regards to major nutrient acquisition and metabolism. The findings reported here will lay a good foundation for understanding the dynamic growth of dinoflagellates during HAB.

## Figures and Tables

**Table 1:** Statistical summary of *A. minutum* unigenes generated using de novo assembly approach.

Statistic	Unigenes
Total unigenes	124,977
GC%	6430
N50 (bp)	1566
Unigenes median lenght (bp)	468
Unigenes mean lenght (bp)	89,669
Total base pairs	112,065,669
BUSCO score	83.5%

**Table 2:** *A minutum* unigenes annotation results against different databases.

Databases	Annotated Unigenes
NCBI-Nr	58,802 (47.05%)
UniProtKB	58,495 (46.80%)
EggNOG	34,602 (27.69%)
KEGG	18,381 (14.70%)
InterPro	57,605 (46.09%)
Gene Ontology (GO)	57,128 (45.71%)

**Table 3:** The identification of the *A. minutum* unigenes encoding homologues for proteins involved in the biosynthesis of cyanobacterial saxitoxins using the blastx with an E value < 0.00001.

Categories	Genes	Putative Function	Unigenes
Core genes	sxtA	Methylation, loading of ACP, Claisen condensation	14
	sxtB	Cyclization	1
	sxtC	Regulatory	-
	sxtD	Desaturation	1
	sxtG	Amidinotransfer	4
	sxtH/T	C-12 hydroxylation	21
	sxtI	Carbamoylation	5
	sxtJ	Regulatory	1
	sxtK	Regulatory	-
	sxtS	Ring formation	8
	sxtU	Short-chain alcohol dehydrogenase	131
	sxtV	Dioxygenase reductase	2
	sxtW	Ferredoxin	10
Modification genes	sxtL	Decarbamoylation	1
	sxtN	Sulfotransferase	1
	sxtO	PAPS biosynthesis	9
	sxtX	*N*-1 hydroxylation	3
Regulatory genes	sxtY	Signal transduction	-
	sxtZ	Signal transduction	-
Transporter genes	sxtF/M	Export of PSTs	4
	sxtP	Binding of PSTs	2
Unknown	sxtE	Unknown	-
	sxtQ	Unknown	-
	sxtR	Unknown	-

## Data Availability

Not applicable.

## Supplementary Materials

The following are available online at https://www.mdpi.com/article/10.3390/biology10090826/s1, Table S1: Quality control and filtering of transcriptome raw reads of *A. minutum*, Table S2: ExN50 profile of *A. minutum* assembled unigenes, Table S3: Annotation of *A. minutum* transcriptome, Table S4: *A. minutum* nitrogen metabolism gene catalogue, Table S5: Blastx result of *A. minutum* unigenes against cyanobacterial saxitoxin biosynthesis genes.
